# Development and validation of a contextual measure of functioning for people living with severe mental disorders in rural Africa

**DOI:** 10.1186/s12888-016-1022-3

**Published:** 2016-09-07

**Authors:** Kassahun Habtamu, Atalay Alem, Girmay Medhin, Abebaw Fekadu, Martin Prince, Charlotte Hanlon

**Affiliations:** 1 Department of Psychiatry, School of Medicine, College of Health Sciences, Addis Ababa University, Addis Ababa, P.O.BOX: 1176 Ethiopia; 2 School of Psychology, College of Education and Behavioral Studies, Addis Ababa University, Addis Ababa, Ethiopia; 3 Aklilu Lemma Institute of Pathobiology, Addis Ababa University, Addis Ababa, Ethiopia; 4 Department of Psychological Medicine, Centre for Affective Disorders, Institute of Psychiatry, Psychology and Neuroscience, King’s College London, London, UK; 5 Centre for Global Mental Health, Institute of Psychiatry, Psychology and Neuroscience, Kings College London, London, UK

**Keywords:** Functioning, Disability, Validation, Sensitivity to change, Factor analysis, Africa, Ethiopia, Mental disorders

## Abstract

**Background:**

Most measures of functioning in people with severe mental disorders (SMD) have been developed in Western societies. Many of the questions in these scales are culture-bound, fail to capture differentiation of tasks by gender and are difficult to adapt to other contexts. The aim of this study was to develop a measure of functioning for people with SMD which is contextually appropriate for a rural African setting.

**Methods:**

A review of existing scales, a qualitative study, free listing and pile sorting exercises, and expert consensus were used to establish a pool of items. Cognitive interviewing guided initial item reduction and refinement. The resulting scale was pilot-tested in people with SMD (*n* = 200) and their caregivers (*n* = 200) to inform further item reduction based on psychometric properties. The final Butajira Functioning Scale (BFS) comprised 33 items that were common to both men and women, and an additional eight items for women only, covering the following domains: self-care, work, and family and community participation. Psychometric properties of the finalized BFS were examined in a facility-based sample of 150 people with SMD and their caregivers (*n* = 150), with longitudinal follow-up of *n* = 84.

**Results:**

The BFS in people with SMD had excellent internal consistency (Cronbach’s α = 0.99), acceptable convergent validity (*r* = 0.88 with the World Health Organization Disability Assessment Schedule [WHODAS-2.0] and *r* = 0.32 with the Brief Psychiatric Rating Scale [BPRS-E]) and was sensitive to change following treatment (effect size =0.50). Addition of the items specific to women did not improve the psychometric properties. The caregiver version had similar psychometric properties but higher mean values for each item and better responsiveness to change. Exploratory factor analysis of the BFS provided evidence of construct validity, with four underlying dimensions.

**Conclusions:**

We have developed a measure of functioning for people with SMD in a rural, low income country setting with acceptable psychometric properties. The BFS is easy to administer, sensitive to changes following treatment and has content, construct and convergent validity. The BFS includes domains from existing measures, but has more emphasis on social and occupational domains, which reflects priorities in the setting.

**Electronic supplementary material:**

The online version of this article (doi:10.1186/s12888-016-1022-3) contains supplementary material, which is available to authorized users.

## Background

Functioning in people with severe mental disorders (SMD) is understood differently by different people, even among clinicians and mental health professionals, because the construct involves different domains and encompasses a wide range of behaviors [1]. Variation exists in the expected socio-cultural roles and their associated functional tasks by gender, in rural vs. urban settings and across cultures. In rural African communities, women are expected to accomplish all domestic tasks and men only are expected to accomplish some community participation tasks, such as involving in conflict resolution. In terms of setting, farming related tasks are common in rural areas whereas trading and public services are rampant in urban areas. Community activities are also expected to be different in different cultural contexts. Generally, functioning has been understood as the capacity of a person to function in different societal roles such as home-maker (or ‘housewife’), worker, student, spouse, family member or friend [2]. Functioning has also been conceptualized as the capacity to work, study, live independently and engage in recreation and romantic life [3–5]. The concept of functioning has also been said to incorporate an individuals’ satisfaction with their ability to meet expected societal roles. However, such conceptualizations of functioning may not be applicable in some socio-cultural contexts. In our previous qualitative study from rural Ethiopia [6], tasks related to self-care, family life, work, interpersonal relationships and participation in community activities were highly valued by family members, neighbors and the community, and considered to be crucial for one’s own survival and the survival of family members. Further, variations are observed among individualistic vs. collectivist societies, whether or not extended family is commonly in the home and among matriarchal vs. patriarchal cultures.

A number of different instruments have been developed to assess functioning, but most originate from Western societies, which are high income, more individualistic and capitalistic [2, 7]. Such scales may lack ecological validity in other socio-cultural settings [2, 8]. Many of the included items are culture- bound and difficult to adapt to other situations, especially in a rural African setting [9]. Furthermore, these scales do not take into account the differentiation of roles by gender which may be more marked in non-Western settings [10] and they fail to present specific tasks important to the local people [11].

The apparently better functional outcomes of people with SMD observed in low-and middle-income countries (LMICs) when compared to high-income countries may be a consequence of measurement bias through use of Western measures [12–14]. There is also an argument that functional recovery is a complex and multi-dimensional concept and meaningful comparison across cultures will not be possible using measures developed in the Western setting [15]. In response, there has been a call for development and validation of contextualised measures of functioning in LMICs, which may also be generalizable to similar settings [13, 15].

The aim of the current study was, therefore, to develop and validate a measure of functional impairment for people with SMD, which is appropriate for a rural African low-income country setting. In doing so, we sought to develop a scale that was socio-culturally relevant, focused on the ability to complete tasks important to the wellbeing of the person and those around him/her and that addressed differences in the roles of men and women.

## Methods

This study has two major components: development of a scale and evaluation of the psychometric properties of the scale. Under this section we described the study setting and this is followed by describing the two components of the study.

### Study setting

The study was conducted in and around the town of Butajira, Gurage zone, Southern Nations, Nationalities and Peoples Region (SNNPR), which is a predominantly rural area. Butajira is located around 135 km south of Addis Ababa, the capital of Ethiopia. There has been a demographic surveillance site (DSS) in Butajira District since 1987 [16], which provides the necessary infrastructure for the conduct of community based epidemiological research studies. Butajira has been the site of a large population-based cohort study of the course and outcome of severe mental disorders (SMD) [17]. The topography in the Butajira area is mixed; it ranges from hot, dry lowlands to cool and mountainous areas [18]. The main occupational functions of the people in the rural areas of Butajira is farming, whereas small scale businesses are common in the town [17]. Maize is the main subsistence grain, with chili pepper being the main cash crop in the lowland parts of Butajira. In the highland areas, *enset* (false banana tree) is the main source of food while khat is the main cash crop [18]. Although much has been done to address gender issues in this area, still women are expected to accomplish all the domestic tasks and support their husbands or parents in different farming activities. At the time of the study, the only mental health services in the Butajira area were being provided by psychiatric nurses in an out-patient clinic of Butajira general hospital.

### Part I: Development of the scale and pilot testing

#### Development of the scale

**Fig. 1:**
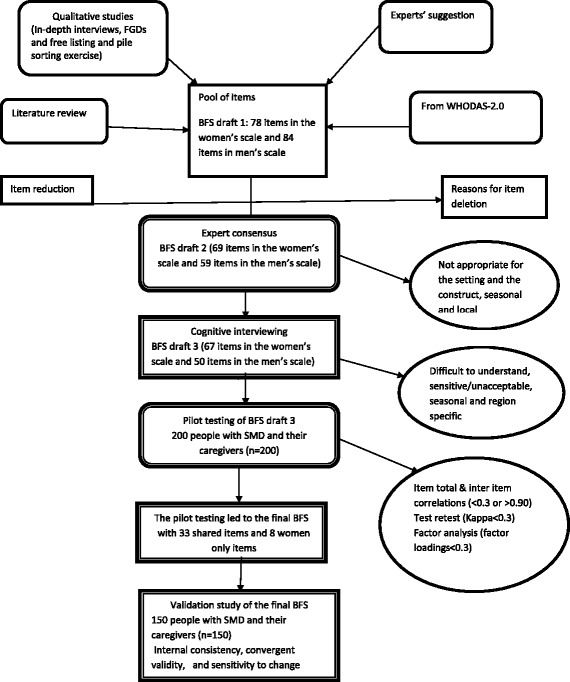
Flow diagram for the procedures followed to develop the BFS

In order to establish the pool of potential items for the new scale, a qualitative study (in-depth interviews and focus group discussions) [6], free listing and pile sorting exercises, review of standard and commonly used measures of functioning and disability and expert consensus were conducted (Fig. 1).

#### Free listing and pile sorting

A free listing and pile sorting exercise was conducted with six group discussions (six individuals in each group). Three of the groups were composed of women and the other three were composed of men. Participants were selected purposively. Project outreach workers (field workers in the SMD course and outcome project) were advised to approach those individuals who had lived in the area for most of their life, who were able to express themselves well in Amharic and represented a range of ages (all aged 18 and above). Efforts were made to make participants representative in terms of education, residence, age and gender. Initially, a free-listing exercise was carried out, asking participants to respond to the following questions; each group only replying with respect to the gender of the participants.

- ‘What are the tasks that men/women must do regularly to care for themselves?’
- ‘What are the tasks that men/women must do regularly to care for their family?’
- ‘What are the tasks that men/women must do regularly to care for their community?’

Once an exhaustive list of tasks has been generated, the facilitator of the group discussions probed using items on the World Health Organization Disability Assessment Schedule (WHODAS-2.0) [19] and any relevant items collected from other scales that had not been mentioned spontaneously. The facilitator asked whether these additional items were relevant for the community and included those which were recognized to be important. A pile-sorting exercise was then carried out using the full list of tasks identified as contextually important in order to identify which items appear to be related to one another, indicating particular domains of functioning. Lastly, participants were asked to rank the tasks in order of importance within the community. Each of the tasks was documented in Amharic, the official language of Ethiopia, using the exact wording agreed by the group, and accompanied by a brief description of each task (Additional file 1: Table S1).

#### Item writing

Each of the tasks in the resulting item pool was translated into scale items following principles of scale construction [20, 21]. Items were framed in a question form like “how much difficulty did you have in accomplishing (…………………..)?” The response categories were a Likert scale, with five options (none, little, moderate, a lot, cannot do task), which are similar to the response categories in the WHODAS-2.0. This formed the first draft of the scale, named the Butajira Functioning Scale draft 1 (BFS draft 1), with 78 items in the women’s scale and 84 items in the men’s scale. There were 47 items common to men and women, 31 for women only and 37 for men only.

#### Expert consensus

A panel of experts, including mental health researchers familiar with the study setting, psychologists and social workers, reviewed each item in the draft scale, and rated them independently in terms of their relevance and representativeness. The expert ratings were analyzed systematically. An expert consensus meeting was conducted over two afternoons and those items that were not relevant to the rural Ethiopian setting, or that measured tasks that were seasonal, insufficiently frequent or highly specific to the locality (e.g. lowlands or highlands) were deleted. Additional items suggested by the expert panel members were also added to the draft scale. This resulted in draft 2 of the scale (BFS draft 2); with 69 items in the women’s scale and 59 items in the men’s scale. There were 37 items common to men and women, 32 for women only and 22 for men only.

#### Cognitive interviewing

Cognitive interviewing [22] was then carried out. In cognitive interviewing, a researcher is expected to ask respondents questions about what they thought each question was asking, whether they could paraphrase each question in their own words and the rationale for their answers [23]. Concurrently, any difficulties with comprehension, together with need for clarifications and any words or expressions that were found to be unacceptable or offensive or sensitive were noted.

In this study, the full list of items derived from the expert consensus meeting was tested using cognitive interviewing in a sample of people with SMD (*n* = 30; 17 males) and their caregivers (*n* = 30; 20 males) in two rounds. In the first round people with SMD were recruited consecutively from the psychiatric out-patient clinic in Butajira general hospital. Poorly understood items that could not be rephrased, and items that were sensitive or unacceptable to respondents were excluded at this stage. This led to draft 3 of the scale (BFS draft 3), with 67 items in the women’s scale and 50 items in the men’s scale. There were 50 items common to men and women, 17 for women only and none for men only. The common items to men and women increased from 37 in Draft 2 to 50 in Draft 3 because the farming related tasks that were expected to be men only were found to be relevant for women also.

### Pilot testing

#### Sample

The Butajira Functioning Scale draft 3 was then pilot-tested in people with SMD (*n* = 200) and their caregivers (*n* = 200) recruited from the Butajira general hospital psychiatric out-patient clinic in order to carry out further item reduction and item refinement. A sub-sample of 50 people with SMD and their caregivers (*n* = 50) were selected randomly to participate in evaluation of test-retest reliability. The inclusion criteria for the pilot study were as follows: a clinical DSM-IV diagnosis of schizophrenia, bipolar disorder or major depressive disorder with psychotic features made by psychiatric nurses, aged 18 years or over, rural residence and able to attend for a follow-up appointment seven days after the initial assessment. The exclusion criteria were participating in the Butajira Course and Outcome of SMD study (to avoid interference in the ongoing study), and presence of a severe co-morbid physical health condition or substance use disorder (as these may limit the participants’ ability to complete self-report measures).

#### Data collection methods

Trained data collectors with 15 years of experience collected the data. Socio-demographic characteristics were measured using a structured questionnaire to collect data on gender, age, education, marital status and relative wealth of both people with SMD and their caregivers. The BFS draft 3 was then administered. Test-retest reliability was evaluated by re-administering the scale within seven days of the original administration.

#### Data analysis

Item reduction was carried out on the basis of a priori criteria. Descriptive statistics (frequency, percentage, mean and standard deviation) were used to examine the distribution of responses to each item. Items which had very low mean values compared to other items, and those which were endorsed or rejected by the vast majority of the respondents were considered for revision or deletion. Items with very low item-scale correlations (<0.3) and very low or very high item-item correlations (<0.3 or >0.90) [24] were either deleted or merged with other items. Test-retest reliability of each item was expressed in terms of intra-class correlation coefficients (ICC). Items which had an ICC lower than 0.3 were either deleted or merged with other items. Exploratory factor analysis (with maximum likelihood extraction and varimax rotation) was used to identify potential number of dimensions, under the sub-scales which emerged from the qualitative studies, and items that load on each of these dimensions. Items which had factor loadings lower than 0.3 and items which had cross loadings (>0.40) were deleted, revised or merged with other items. This led to the final version of the scale (the BFS), comprising 33 shared items, 8 items specific to women and no items specific to men (see Additional file 1: Table S6).

### Part II: Validation study

A facility-based cohort study was conducted to evaluate the psychometric properties of the finalized BFS.

#### Sample

New or acutely relapsed cases of people with SMD and their caregivers were recruited from the Butajira general hospital psychiatric out-patient clinic. In order to detect a correlation coefficient of 0.8 between two continuous measures (the BFS and the Amharic version of the WHODAS-2.0), with margin of error of 0.1, with alpha = 0.05 and power of 80 %, a sample of (*n* = 118) persons were required (calculated using G* Power software [25]. The change in score on the two functioning measures (BFS and WHODAS-2.0) that is considered to be a meaningful indicator of improvement was unknown. Therefore, in order to calculate the required sample size, the effect size of the change in mean scores between the two time points was taken into account. In order to be able to detect a standardized effect size 0.6 (moderate to large), with 80 % power and alpha = 0.05, to ensure that the standardized effect size was not as low as 0.3 (small), a sample size of 90 persons was required (calculated using G* Power software) [26].

The inclusion criteria included DSM-IV diagnosis of schizophrenia, bipolar disorder or psychotic depression made by psychiatric nurses, new onset or in acute relapse, age 18 or over and able to attend for a follow-up appointment six weeks after the initial assessment. The exclusion criteria were severe co-morbid physical health condition and substance use disorder (as these may limit the participants’ ability to complete self-report measures).

#### Measures

The BFS was administered, together with the Amharic version of the WHODAS-2.0 and the expanded version of the Brief Psychiatric Rating Scale (BPRS-E).

*World Health Organization Disability Assessment Schedule* (*WHODAS*- *2.0*): The WHODAS was developed as a cross-cultural measure of the difficulty of daily activities and social participation experienced by a person in the past 30 days [27]. It is a generic instrument, not aimed at specific populations or specific health conditions [28]. The WHODAS 2.0 is available in different forms (12 and 36 item versions, self-administered or interviewer administered and responded by patient, caregiver and clinician) [28–30] and its validity and reliability have been tested in a number of studies [28, 30–34]. The WHODAS-2.0 has been adapted and validated in different languages and cultures, but limited to high-income and middle-income countries [28, 30, 35].

*Brief Psychiatric Rating Scale* (*BPRS-E*): It is a 24-item observer-rated symptom scale covering four domains of symptoms of SMD (positive symptoms, negative symptoms, anxiety and depressive symptoms, and manic excitement or disorganization) and gives an overall indication of clinical symptom severity. The BPRS-E has been used widely to detect clinical improvement in response to an intervention [36] and has been used previously in Ethiopia [37].

*Socio-demographic questionnaire:* A socio-demographic characteristics questionnaire was used to collect data on the gender, age, education, marital status and relative wealth of both people with SMD and their caregivers. Moreover, the diagnosis of each patient was traced from the patient’s cards.

#### Data analysis

Convergent validity was assessed by calculating Pearson’s correlation coefficient (*r*) for the association between BFS score and scores on the WHODAS- 2.0 and BPRS-E. Internal consistency was assessed by calculating Cronbach’s alpha.

In order to evaluate the sensitivity to change of the BFS over time, both internal and external responsiveness were determined in line with recommended practice [38]. Internal responsiveness is the change in a measure over time and was evaluated by paired sample t-test, effect size (change in mean divided by standard deviation of the baseline score) and the standardized response mean (SRM), which is calculated by dividing the change in mean score by the standard deviation of the change scores (Δ mean/Δ SD). External responsiveness is the extent to which change in the index measure (the BFS) corresponds to change in an external, reference measure (the BPRS-E) [38]. Spearman rank order correlation of the change scores from the two measures was computed to determine external responsiveness to change.

### Ethical considerations

Ethical approval was obtained from the College of Health Sciences Institutional Review Board, Addis Ababa University. Written informed consent was obtained from most patient participants and from all of the caregivers after the nature of the study and the information sought had been fully explained. For a few patient participants, who were acutely unwell, we either obtained permission from their guardians or got written consent after their condition was improved during the follow-up assessment.

## Results

### Free listing and pile sorting

Equal number of males and females (18 in each gender) participated in the free listing and pile sorting exercise. The mean age of male and female participants was 31.0 and 26.4 years, respectively. In terms of religion, 17 were Christian and 19 were Muslim. All of the participants reported farming as their main occupation.

Male participants grouped the specific activities/tasks they identified into the following domains: self-care, farming, family life, social participation, religious activities, trading and entertainment. Women grouped the tasks or activities they identified into similar domains, but with an additional domain of domestic tasks and no domain for entertainment. Both men and women respondents emphasized that tasks related to work, family, community participation and caring for oneself are particularly critical in the setting (Additional file 1: Table S1). When asked to rank order the domains and the specific tasks, participants across all groups placed farming activities at the top followed by domestic tasks, and then taking care of partners and children, participating in different community activities and finally caring oneself.

### Pilot testing

#### Socio-demographic characteristics of participants

A total of 200 people with SMD (68 % male) and 200 caregivers (59 % male) participated in the pilot study. The details of the socio-demographic characteristics of the participants are presented in Table 1. Almost half of the people with SMD had schizophrenia (53 %) and the remaining had either bipolar disorder (28 %) or depressive disorder (19 %) with psychotic features.

**Table 1:** Socio-demographic characteristics of participants

Characteristics		Pilot study		Validation study	
		Service users (*n* = 200) *N* (%)	Caregivers (*n* = 200) *N* (%)	Service users (*n* = 150) *N* (%)	Caregivers (*n* = 150) *N* (%)
Sex	Male	124 (68.0)	118 (59.0)	81 (54.0)	121 (80.7)
	Female	76 (32.0)	82 (41.0)	69 (46.0)	28 (18.7)
Age	Mean (SD)^a^	30.5 (10.90)	36.8 (13.26)	30.4 (10.04)	35.1 (12.14)
Ethnicity	Gurage	109 (54.5)	108 (54.0)	60 (40.0)	58 (38.7)
	Silti	77 (38.5)	77 (38.5)	80 (53.3)	82 (54.6)
	Others	14 (7.0)	15 (7.5)	10 (6.7)	10 (6.7)
Religion	Orthodox Christian	38 (19.0)	40 (20.0)	27 (18.0)	25 (16.7)
	Muslim	147 (73.5)	148 (74.0)	118 (78.7)	120 (80.0)
	Protestant	15 (7.5)	12 (8.0)	5 (3.3)	5 (3.3)
Marital status	Single	92 (46.0)	46 (23.0)	53 (35.3)	34 (22.7)
	Married	93 (46.5)	140 (70.0)	81 (54.0)	112 (74.7)
	Divorced	2 (1.0)	1 (0.5)	4 (2.7)	0 (0.0)
	Separated	6 (3.0)	2 (1.0)	7 (4.7)	0 (0.0)
	Widowed	7 (3.5)	11 (5.5)	5 (3.3)	3 (2.0)
Education	Can’t read and write	58 (29.0)	54 (27.0)	57 (38.0)	34 (22.7)
	Read and write only	68 (34.0)	83 (41.5)	41 (27.3)	57 (38.0)
	Primary school	55 (27.5)	37 (18.5)	40 (26.7)	42 (28.0)
	Secondary school	12 (6.0)	17 (8.5)	10 (6.7)	10 (6.7)
	Post -secondary	6 (3.0)	9 (4.5)	2 (1.3)	6 (4.0)
Occupation	Farming	148 (74.0)	162 (81.0)	77 (51.3)	109 (72.7)
	Trading	8 (4.0)	6 (3.0)	12 (8.0)	13 (8.7)
	Government employee	3 (1.5)	6 (3.0)	1 (0.7)	8 (5.3)
	Student	9 (4.5)	9 (4.5)	9 (6.0)	6 (4.0)
	House wife	19 (9.5)	13 (6.5)	27 (18.0)	12 (8.0)
	No employment	11 (5.5)	2 (1.0)	22 (14.7)	1 (0.7)
	Others	2 (1.0)	2 (1.0)	2 (1.3)	1 (0.7)
Relative wealth	Less	107 (53.5)	92 (46.0)	67 (44.7)	42 (28.0)
	More	4 (2.0)	1 (0.5)	4 (2.7)	4 (2.7)
	Equal	89 (44.5)	107 (53.5)	79 (52.7)	104 (69.3)
Diagnosis	Schizophrenia	106 (53.0)	–	68 (45.3)	–
	Bipolar disorder	56 (28.0)	–	41 (27.3)	–
	Major depressive disorder	38 (19.0)	–	41 (27.3)	–

^a^Standard deviation

#### Psychometric properties of the pre-finalized scale

There were no items that were endorsed or not endorsed by all respondents. Nevertheless, the responses (particularly the responses of service users) were skewed to the right; that is towards less severe disability. Few items were found to have exceptionally high or low mean values compared to the mean values of all other items in the sub-scale (Additional file 1: Table S2). All items had acceptable levels of item-scale correlation (*r* > 0.3). Most of the items had item-total correlations which ranged from 0.60 to 0.78. Around 15 items had item-scale correlations of 0.80 to 0.90. There were only two items which had <0.60 item-scale correlation. Item-scale correlations for most of the items were similar in both service users and caregivers.

No item had an inter-item correlation <0.30 and most of the items had item-item correlation >0.50. There were items with an item-item correlation >0.90: working in the field (ploughing, reaping), working in the field (weeding, digging, threshing, cleaning land for threshing), following up the wellbeing of the livestock, collecting grass and straw for livestock food, availing water for the livestock or taking them to water, and looking after livestock during the day. Items preparing food/cooking, preparing coffee, cleaning the house, cleaning cooking and serving utensils, able to keep cooking and serving utensils in order and fetching water, which are all household tasks supposed to be accomplished by women, were highly correlated with each other (*r* > 0.90). Items related to taking care of children, which included bathing children (keeping children’s hygiene), following up children’s hygiene, advising and disciplining children, feeding children, supporting children in wearing clothes, changing children’s clothes on time and able to keep children from danger had also inter-item correlation >0.90.

Most of the items had test-retest reliability >0.30 for both service users and caregivers. Only four items had ICC <0.30 in both the service users’ and caregivers’ data: able to eat food in a proper manner, brushing teeth, able to keep oneself from danger and working in the field (ploughing, reaping). Nevertheless, there were relatively more number of items with test-retest reliability <0.30 in the caregiver data (Additional file 1: Table S2). It appeared that items in the self-care domain had lower test-retest reliability than items in all other domains. Overall, higher test-retest reliability was found among service users than caregivers.

#### Factor analysis

Factor analysis of the self-care sub-scale identified two factors (the eating factor and the hygiene factor), both in the service users’ and caregivers’ data (Additional file 1: Table S5). Two items relating to eating loaded separately to the other ten items (relating to hygiene).

Factor analysis of the men and women shared work items identified a one factor solution in both the service users’ and caregivers’ data, which accounted for 72.8 % and 73.8 % of the variance among service users and caregivers, respectively. The women only work items, on the other hand, loaded onto two factors. Thirteen items loaded on a farming factor and the remaining 11 items loaded on the domestic tasks domain.

Factor analysis of both the men and women shared and the women only social functioning items showed the presence of two factors (see Additional file 1: Table S5): (1) family and children and (2) community participation. In the shared social functioning items, nine of the items loaded onto the family and children factor and 17 of the items on the community participation factor. In the women only social functioning items, 14 items loaded onto the family and children factor, whereas 17 items loaded onto the community participation factor. Items that were presented to only women participants in the social functioning domain loaded clearly onto the family and children factor. Items at the family level and the community level had clear loadings, whereas items at the neighborhood level had cross loadings, especially in the caregivers’ data.

### Validation study

One hundred and fifty people with SMD (54 % male) and 150 caregivers (80.7 % male) participated in the validation study. The socio-demographic characteristics of these participants were similar to the pilot study participants (see Table 1).

#### Descriptive statistics and internal consistency

The frequency distributions of the scores in the overall scale and the sub-scales were nearly normal in both the service user and caregiver data. The mean scores of each sub-scale and the overall BFS among service users and caregivers were as follows (Table 2): Self-care (8.0, 12.9), shared work items (12.7, 16.8), women only work items (18.4, 24.9), social functioning (25.4, 37.6), overall BFS (51.8, 75.5). Mean scores across all the sub-scales and the overall BFS were higher in the caregiver data than in the service user data. The minimum and maximum observed scores in the sub-scales and the overall scale were: Self-care (0, 36), shared work items (0, 28), women only work items (0, 60), overall BFS (0, 151). Internal consistency was above 0.90 (excellent) for all sub-scales and the overall scale. The overall BFS had internal consistency of 0.99. The internal consistency of subscales ranged from 0.94 to 0.98 (Additional file 1: Table S3)).

**Table 2:** Overall scale and sub-scales’ descriptive statistics of the validation study data

	Service users				Caregivers			
Scale	Maximumpossible score	Minimum observed score	Maximum observed score	Mean (SD)	Maximum possible score	Minimum observed score	Maximum observed score	Mean (SD)^a^
Self-care	36.0	0	36.0	8.0 (9.4)	36.0	0	36.0	12.9 (11.3)
Work (shared items)	28.0	0	28.0	12.7 (9.2)	28.0	0	28.0	16.8 (8.8)
Work (women only)	60.0	0	60.0	18.4 (15.4)	60.0	0	60.0	24.9 (17.3)
Social functioning	68.0	0	68.0	25.4 (21.5)	68.0	0	68.0	37.6 (21.5)
Overall BFS	164.0	0	152.0	51.8 (42.1)	164.0	0	162.0	75.5 (44.0)

^a^ *SD* standard deviation

#### Convergent validity

The correlation between the sub-scales’ and the overall scale scores of the BFS and the WHODAS-2.0 and BPRS-E are presented in Additional file 1: Table S4 and Table 3, respectively. As expected, a high correlation coefficient was found between the BFS and the WHODAS-2.0, indicating that the two scales are measuring the same construct. The highest correlation coefficients were found between specific domains measuring similar characteristics in the two measures, such as the BFS and WHODAS sub-scales of self-care, work and social participation. Inter-correlations between the overall scale and the different domains of the BFS and the WHODAS-2.0 ranged from 0.55 to 0.88 among service users and 0.36 to 0.89 among caregivers.

**Table 3:** Pearson’s correlation between BPRS-E and the BFS (*n* = 150)

Scale	Service users		Caregivers	
	Baseline	Follow-up	Baseline	Follow-up
Self-care	0.27	0.49	0.24	0.49
Work (shared)	0.17	0.42	0.26	0.41
Work (women only)	0.13	0.41	0.20	0.41
Social functioning	0.28	0.54	0.37	0.53
Overall scale	0.25	0.53	0.32	0.52

Positive correlations were found between the overall scale and all the domain scores of the BFS and BPRS-E scores, both at baseline and follow-up. However, correlation coefficients were found to be weak (0.13 to 0.28 among service users and 0.24 to 0.37 among caregivers) at baseline and moderate at follow-up (0.41 to 0.54 among service users and 0.41 to 0.53 among caregivers). As expected, as symptom scores increased, there was an increase in disability scores, indicating the convergent validity of the BFS.

#### Sensitivity to change

The scores in the overall BFS and in the sub-scales were reduced after treatment for six weeks. The changes were statistically significant, among both the service users and the caregivers, using the paired sample t-test. However, the effect sizes (ES I) and standardized response means (ES II) were small as reported by the service users and moderate as reported by the caregivers (Table 4). Overall, the BFS is sensitive to small changes in clinical symptoms resulting from treatment. However, the inclusion of women only items in the work sub-scale, which are related to domestic tasks, did not improve the sensitivity to change of the BFS in general or the work sub-scale in particular.

**Table 4:** Internal sensitivity to change of the BFS (*n* = 84)

Scale	Service users				Caregivers			
	Difference scores		ES^a^	SRM^b^	Difference scores		ES	SRM
	Mean	SD			Mean	SD		
Self-care	2.1	10.68	0.20	0.19	4.4	10.81	0.36	0.40
Work (shared)	2.0	10.44	0.21	0.19	3.2	10.13	0.37	0.32
Work (women only)	3.6	15.93	0.21	0.22	5.6	18.12	0.32	0.31
Social functioning	4.8	23.97	0.21	0.20	12.7	22.90	0.59	0.55
Overall scale	10.4	46.07	0.22	0.23	22.6	46.65	0.50	0.49

^a^Effect size; ^﻿b﻿﻿﻿^Standardized response mean﻿﻿

In terms of external responsiveness, the BFS and BPRS-E scores co-varied (Table 5). That is change in symptom severity scores are accompanied by change in scores of functional impairment.

**Table 5:** External sensitivity to change of the BFS (*n* = 84)

Scale	Spearman’s correlation	
	Service users	Caregivers
Self-care	0.21	0.24
Work (shared items)	0.30	0.36
Work (women only)	0.28	0.33
Social functioning	0.41	0.37
Overall scale	0.37	0.36

#### Factor analysis

Exploratory factor analysis of items shared between men and women in the finalized BFS identified factors corresponding to concepts of functioning obtained in the qualitative study, free listing and pile sorting and the pilot study: self-care (9 items), work (7 shared items for men and women and 8 women only items), and social functioning (17 items) (Table 6).

**Table 6:** Factor loadings of the men and women shared items in the validation study (*n* = 150)

Item	Factor 1	Factor 2	Factor 3
Able to ask for or prepare and eat food when needed		0.59	
Washing own body		0.79	
Washing hands before and after eating		0.78	
Washing own clothes		0.69	
Cutting nails		0.78	
Able to change clothes when it gets dirty	0.43	0.80	
Able to keep oneself from danger		0.63	
Using the toilet properly		0.63	
Washing hair	0.43	0.81	
Working in the field			0.82
Kitchen gardening			0.82
Looking after and attending livestock during the day			0.81
Cutting grass			0.80
Splitting firewood			0.83
Going to market	0.42		0.71
Travelling for one hour			0.48
Following up children’s health	0.64	0.42	0.45
Motivating and encouraging children in their education and other activities	0.66		0.45
Communicating well (living in peace and agreement) with family	0.69		
Discussing family issues with family members	0.75		
Helping parents or close elderly relatives	0.77		
Maintaining social contact with relatives	0.73		
Following up children’s hygiene	0.73		0.41
Advising and disciplining children	0.78		
Communicating well (living in peace and harmony) with neighbors	0.77		
Doing different tasks in cooperation with neighbors	0.74		0.42
Going and attending when there is mourning in the neighborhood	0.74		
Participating in *Idir* ^a^	0.71		
Visiting postnatal women, people who are sick, prisoners and elderly	0.72		
Participating in and preparing *Mahiber/Senbete/Lika/Dado* ^b^	0.66	0.43	
Attending *Kebele* ^c^ and village meetings	0.65		0.40
Going to church/mosque	0.64		
Giving food or money for those who are in need	0.60		

^a^Local funeral insurance group

^b^Local religious organizations for Christians and Muslims

^c^The lowest administrative structure in Ethiopia (sub-district)

## Discussion

We have developed a measure of functional impairment for people with SMD, which is contextually appropriate for a rural African setting and has acceptable psychometric properties. Our study also shows that adding women only items in the scale do not bring about improvements in the psychometric properties of the scale, but factor analysis indicated that these items loaded separately to the other work items and our qualitative studies indicate that domestic tasks may be useful to assess improvement of the life of women with SMD in the clinical setting. Our qualitative study [6] and the free listing and pile sorting exercise enabled us to identify the broad domains of functioning and the specific daily activities that an adult person is expected to accomplish in a rural low-income country setting. Participants emphasized that these activities are crucial for the survival of both the person and the people around him/her. It appears that men and women are expected to accomplish a number of similar tasks, except domestic tasks, which are left only to women. Women are required to accomplish almost all tasks that men are to accomplish, but men are not required at all to engage in domestic tasks.

The domains of functioning we identified are similar to the domains found in various cross-cultural measures of functioning [39, 40], including the WHODAS [29], although some domains of functioning such as mobility and understanding were not prioritized in our study. However, the specific activities in each domain are less generalizable, and are relevant to the context where the study was conducted. They are directly or indirectly crucial for the survival of both the person and his/her family members. Our finding of daily functional activities that are relevant to the local situation in rural Ethiopia (and similar agricultural communities across Africa) and show differentiation by gender is consistent with the ideas of Bolton and Tang [11], who said that functional tasks vary greatly according to sex, culture and environment. Nevertheless, in our study, the gender specific items did not improve the psychometric qualities of the scale we developed.

In the pilot study, the endorsement of items from both service users and caregivers were well-distributed. There were no items endorsed by everyone or not endorsed by any participants. Overall, responses were skewed to the right, which is expected as we recruited people with SMD who were stabilized taking medication for some time. Few items were found to have mean values higher or lower than all the other items in the sub-scale they belong to. This is logical when we see these items taking the context into account. For example, farming related activities such as ploughing and harvesting are known to be the most difficult tasks in rural areas. Using the toilet properly is found to be the easiest of all items, which sounds correct in people with SMD who are stabilized, though this task may be more difficult in people who are actively psychotic.

All items performed well in terms of item-total correlation. Only two items had item-total correlation <0.60 and the lowest item-total correlation is 0.47. However, there are quite a number of items with item-item correlation >0.90, though there are no items with item-item correlation <0.30. Those items with item-item correlation >0.90 were considered for merging, deletion, or modification. The majority of items performed well with regard to test-retest reliability but four items were found to have test-retest reliability <0.30 among both service users and caregivers and these items were considered for revision or deletion. Overall, items were found to have better test-retest reliability for service users compared to caregivers.

We carried out exploratory factor analysis using pilot study data for each of the sub-scales separately, to identify the items which load onto the dimensions identified. In the self-care sub-scale, two items loaded onto the eating factor and ten of the items loaded onto the hygiene factor. We found that the items related to eating (able to eat food in a proper manner and able to eat food on time) were understood as appetite and availability, which do not indicate an individual’s ability to care for him/herself. Hence, these two items were merged and rewritten as “able to ask for or prepare and eat food when needed.” Nevertheless, it is logical and acceptable for items related to eating and related to hygiene loaded separately. In the work sub-scale, we found one factor solution for the men and women shared items, but in the women only items we found two factor solution (farming and domestic tasks). It is striking that the different farming and domestic tasks loaded differently. In rural Ethiopia, all the domestic tasks are expected to be accomplished by women. In addition, women are expected to support their husbands or their parents in all aspects of farming.

Factor analysis of the social functioning items, both the shared items and the woman only items, resulted in two factors (family and children and community participation), although there were items which cross-loaded. This clearly reflects the reality in rural Ethiopia, where adult people are not only expected to take care of their family members (parents, children and siblings), but are also expected to participate in different kinds of community activities such as *“Idir”* [local self-help group), weddings, funerals, meetings and in other social gathering and developmental activities.

Overall, the factors we identified through exploratory factor analysis are consistent with the findings of our qualitative study [6] and the free listing and pile sorting exercise. In addition, they are more or less similar with the domains in the WHODAS [29, 41, 42], though the specific activities under each domain are different.

Our validation study showed that the new functioning measure that we have developed has excellent internal consistency. It has positive and strong correlation with WHODAS-2.0 indicating that the two instruments measure the same construct. The new measure has also been demonstrated to have a positive correlation with symptom severity, both at baseline and follow-up. Previous studies showed that there is a positive correlation between symptom severity and functional impairment [9, 43–45]. Nevertheless, in our study, the correlation between symptom severity and functional impairment was found to be higher at follow-up than at baseline. This may be due to low variability of scores at baseline. We included new presentations and people in a state of relapse and both symptom and functioning scores were found to be high. Low or moderate correlation between symptom severity and functional impairment is expected. We found in our qualitative study [6] that functional impairment in people with SMD is the result of not only symptoms related to the illness but also a multitude of other factors related to the patient, family and the socio-economic condition.

Our new measure has the ability to detect changes overtime. We found statistically significant mean changes in functioning scores after six weeks of treatment of new cases and cases in relapse. When we see the changes in terms of effect size and SRM, they are small among service users and moderate among caregivers. The small effect size among service users may be due to under reporting of their functional impairment [46] and lack of their capacity to evaluate themselves as a result of active symptoms [47]. The positive and statistically significant correlation between the change scores of symptom severity and functional impairment indicated that change in symptom severity is accompanied by change in functional impairment. This gives evidence to the convergent validity of our new measure.

Our qualitative study and the free listing and pile sorting exercise clearly revealed that women are expected to accomplish all aspects of the domestic tasks in addition to supporting men in farming related activities. So, including domestic tasks in the scale and having separate versions for men and women is useful to have the full picture of the functioning of women with SMD. Nevertheless, our validation study showed that the women specific items have the same properties as the shared items in terms of both convergent validity and responsiveness to change. The women specific work items have also moderate or strong correlation with the shared work items. These all indicate that for quick epidemiological population based large surveys, one can omit the women specific items and use only men and women shared items. When one needs to know all aspects of the functioning of women with SMD and to asses improvement in routine clinical practice, it would be wise to include the women specific items in the scale and have separate versions for men and women.

The strengths of our study are that we followed rigorous procedures to develop and validate the new measure; and the study is nested in a big longitudinal research project. One limitation of our study is that in the validation study, the WHODAS and the BFS were administered one after the other with no time gap in between. Therefore, the responses to the items of the first scale might have influenced the responses to the items in the next scale. A number of studies have indicated that functional improvement after treatment, in people with SMD, lag behind symptom changes [48, 49]. So, our six weeks follow-up period may not be enough to evaluate the extent to which the new functioning measure can pick meaningful changes.

## Conclusions

We have developed a measure of functioning in people with SMD, with items relevant to the local context in rural low-income settings, that is easy and fast to administer, has very good construct validity and excellent internal consistency and is sensitive to changes in clinical state over time. We recommend that future research should be targeted at adapting and checking validity and reliability of the BFS in other parts of rural Ethiopia in particular and in other parts of rural Africa in general. It will also be invaluable to study the sensitivity to change of the scale with a longer period of follow-up.

## Additional file

Additional file 1: Table S1. Summary of the domains and specific activities identified in the free listing and pile sorting exercise. **Table S2.** Mean value, item-scale correlation and test-retest reliability of items in the pilot study. **Table S3.** Mean, item –scale correlation and alpha value of items in the validation study. **Table S4.** Pearson’s correlation between WHODAS and the BFS (n = 150): service users (caregivers). **Table S5.** Exploratory factor analysis of the piloting data. **Table S6.** English version of the Butajira Functioning Scale (BFS). (DOCX 97 kb)

## Abbreviations

BFS: Butajira functioning scale
BPRS: Brief psychiatric rating scale
DSM: Diagnostic and statistical manual for mental disorders
DSS: Demographic surveillance site
ES: Effect size
LMICs: Low and middle income countries
SMD: Severe mental disorders
SNNPR: Southern Nations, Nationalities and People’s Region
SRM: Standardized response mean
WHODAS: World Health Organization Disability Assessment Schedule
