# Supplementary material for: Development and validation of a contextual measure of functioning for people living with severe mental disorders in rural Africa
Source: BMC Psychiatry. 2016 Sep 7;16(1):311. doi: 10.1186/s12888-016-1022-3 (PMC5015207; doi:10.1186/s12888-016-1022-3)
Supplement: Additional file 1: Table S1. — Summary of the domains and specific activities identified in the free listing and pile sorting exercise. Table S2. Mean value, item-scale correlation and test-retest reliability of items in the pilot study. Table S3. Mean, item –scale correlation and alpha value of items in the validation study. Table S4. Pearson’s correlation between WHODAS and the BFS (n = 150): service users (caregivers). Table S5. Exploratory factor analysis of the piloting data. Table S6. English version of the Butajira Functioning Scale (BFS). (DOCX 97 kb) [file 12888_2016_1022_MOESM1_ESM.docx]

**Additional file 1. Summary of the domains and specific activities identified in the free listing and pile sorting exercise.**

| Domain | Specific activity | |
| --- | --- | --- |
|  | **Men** | **Women** |
| Self-care (health) | Feeding self on time | Able to feed self |
|  | Having balanced diet | Able to eat food on time and with no reminder |
|  | Eating food that is comfortable to oneself | Wash self |
|  | Protecting self from danger | Wash clothes |
|  | Wash self | Changing clothes on time and with no reminder |
|  | Wash clothes | Protecting self from danger |
|  | Cutting nails | Using family planning  Cleaning the house and the surrounding |
|  | Getting haircut | Entertaining self |
|  | Brushing teeth |  |
|  | Building latrine |  |
|  | Using mosquito net |  |
|  | Building a separate house for cattle |  |
|  | Changing clothes on time and with no reminder |  |
| Farming (tasks related to farming) | Ploughing | Kitchen gardening |
|  | Sowing | Preparing “Inset” or false banana |
|  | Weeding | Sowing when it is done in line |
|  | Digging | Digging |
|  | Using weedicides and insecticides | Weeding |
|  | Reaping | Making the land flat when “teff” (locally grown and staple crop) is sown |
|  | Threshing | Looking after cattle |
|  | Planting “Inset” or false banana tree | Cutting grass |
|  | Kitchen gardening | Rearing animals |
|  | Harvesting pepper (gathering and sorting) | Making cattle’s food ready |
|  | Planting and caring trees | Cleaning the land for the purpose of threshing |
|  | Rearing cattle | Helping in harvesting |
|  | Raising chicken | Working as daily labourer |
|  | Bee farming |  |
|  | Splitting wood |  |
|  | Looking after cattle |  |
|  | Tethering and untethering cattle |  |
|  | Cutting grass |  |
|  | Building fence |  |
|  | Working as daily labourer |  |
| Domestic tasks |  | Cooking/preparing food |
|  |  | Preparing coffee |
|  |  | Cleaning the house |
|  |  | Fetching water |
|  |  | Tidying house utensils |
|  |  | Going to mill house to get grain ground |
|  |  | Cleaning house utensils |
|  |  | Washing clothes |
|  |  | Tethering and untethering cattle |
|  |  | Cleaning the animal area |
|  |  | Preparing local beverages |
|  |  | Doing handicrafts |
|  |  | Splitting firewood |
| Domain | **Specific activities** |  |
|  | **Men** | **Women** |
| Family life (marriage and children) | Building house | Getting married |
|  | Getting married | Having children |
|  | Having children | Feeding and dressing children |
|  | Feeding and dressing children | Washing children |
|  | Following up children’s health and hygiene | Following up children’s health |
|  | Buying clothes for children | Washing children’s clothes |
|  | Advising and disciplining children | Changing children’s clothes on time |
|  | Motivating and encouraging children | Sending children to school |
|  | Sending children to school | Covering children’s educational expenses |
|  | Covering the educational expenses of children | Making educational materials ready for children |
|  | Making educational materials ready for children | Following up the educational performance of children |
|  | Giving love for and entertaining children | Arranging time for children to study |
|  | Discussing with family members about family issues, plans and goals | Protecting children from danger |
|  | Making necessary things at home ready | Giving love for and entertaining children |
|  | Helping and supporting parents | Discussing issues with children |
|  | Helping children to be independent | Caring for husband |
|  | Covering expenses during holidays |  |
|  | Arranging parties and inviting relatives |  |
|  | Splitting firewood |  |
| Social life (social participation) | Attending coffee ceremonies with neighbours | Attending coffee ceremonies with neighbours |
|  | Communicating well with family members, neighbors and the community | Communicating well with family members, neighbors and the community |
|  | Doing different tasks in cooperation with neighbours | Contributing (money and grain) to “Idir” |
|  | Keeping security (peace) in the neighbourhood | Participating in “Idir” activities |
|  | Going to weddings, funerals, birth day celebrations and other celebrations | Doing different tasks in cooperation with neighbours |
|  | Contributing (money and grain) to “Idir” or a funeral insurance group | Supporting neighbours in different tasks when there is mourning or any other ceremony |
|  | Doing different tasks in “Idir” | Going and attending when there is mourning in the neighbourhood |
|  | Attending “Idir” meetings | Giving gift to neighbours when there is mourning, wedding, birth day celebration or any other ceremony |
|  | Working as executive committee member in “Idir” | Inviting neigbours during holidays |
|  | Visiting post-natal women, people who are sick, prisoners, elders and relatives | Going to weddings, funerals, and birth day celebrations |
|  | Participating in development activities | Visiting post-natal women, people who are sick, elderly and relatives |
|  | Making contribution to development activities | Mediating those who are in conflict |
|  | Participating in “Mahiber”/”Senbetie”/”Lika”/”Dado” (they all are local religious organizations) | Attending sub-district and village meetings |
|  | Participating in Kebele (sub-distrct) and village meetings | Participating in elections |
|  | Working as executive committee member (playing leadership role) in the community |  |
|  | Helping the elderly |  |
|  | Helping (supporting) orphans |  |
|  | Cooperate and contribute to building church/mosque |  |
|  | Participating in and having saving group |  |
| Domain |  | **Specific activities** |
|  | **Men** | **Women** |
| Religious activities (Religious participation) | Going to church/mosque  Praying  Fasting | Going to church/mosque  Praying  Fasting |
|  | Contributing (money or material) to building church/mosque  Attending religious teaching  Implementing religious rules  Performing religious marriage  Giving  Participating in and preparing “Senbetie”/”Mahiber”/”Lika”/”Dado”  Coordinating, participating and supporting religious celebrations  Giving food to the poor (“Sedeka”) | Participating in and preparing “Senbetie”/”Mahiber”/”Lika”/”Dado”  Celebrating religious holidays |
| Trading (Doing business) | Trading grain, pepper, cattle and other different kinds of commodities | Trading grain, sugar, gas, salt, soap, and other different kinds of commodities |
|  | Going to market | Saving |
|  | Saving |  |
|  | Traveling long distance |  |
| Entertainment | Visiting one’s farm land |  |
|  | Preparing for and celebrating holidays |  |
|  | Chewing khat in group |  |
|  | Taking alcoholic drinks |  |
|  | Going to the town |  |
|  | Talking (enjoying) with friends |  |
|  | Listening radio |  |
|  | Watching television |  |
|  | Reading |  |

**Additional file 2. Mean value, item-scale correlation and test-retest reliability of items in the pilot study.**

| Item | Service users (N=200) | | | | Caregivers (N=200) | | | |
| --- | --- | --- | --- | --- | --- | --- | --- | --- |
|  | **Mean** | **Item-scale correlation** | | **ICC*** | **Mean** | **Item-scale correlation** | **ICC** | |
|  |  |  | |  |  |  |  | |
| Self-care | | | | |  | | | |
| Able to eat food in a proper manner | 0.59 | 0.65 | | 0.24 | 0.73 | 0.55 | 0.17 | |
| Able to eat food on time | 0.54 | 0.69 | | 0.43 | 0.82 | 0.67 | -0.06 | |
| Washing own body | 0.58 | 0.75 | | 0.44 | 0.88 | 0.83 | 0.07 | |
| Washing hands before and after eating | 0.44 | 0.80 | | 0.55 | 0.76 | 0.84 | 0.12 | |
| Washing own clothes | 0.74 | 0.76 | | 0.46 | 1.12 | 0.83 | 0.07 | |
| Cutting nails | 0.58 | 0.81 | | 0.53 | 0.97 | 0.89 | 0.25 | |
| Washing hair | 0.57 | 0.84 | | 0.53 | 1.05 | 0.92 | 0.22 | |
| Getting haircut (Getting hair dressed) | 0.62 | 0.82 | | 0.59 | 1.07 | 0.92 | 0.29 | |
| Brushing teeth | 0.56 | 0.85 | | 0.20 | 1.04 | 0.90 | 0.28 | |
| Able to change clothes when it gets dirty | 0.59 | 0.74 | | 0.43 | 1.09 | 0.84 | 0.17 | |
| Able to keep oneself from danger | 0.52 | 0.67 | | 0.22 | 0.84 | 0.74 | 0.13 | |
| Using the toilet properly | 0.32 | 0.68 | | 0.32 | 0.52 | 0.58 | 0.04 | |
| Work | | | | |  | | | |
| Working in the field (ploughing, reaping) | 1.71 | 0.81 | | 0.18 | 2.24 | 0.79 | | 0.15 |
| Working in the field (weeding, digging, threshing, cleaning land for threshing) | 1.68 | 0.84 | | 0.37 | 2.22 | 0.80 | | 0.18 |
| Kitchen gardening | 1.32 | 0.88 | | 0.35 | 1.80 | 0.89 | | 0.42 |
| Collecting grass and straw for livestock food | 1.15 | 0.93 | | 0.36 | 1.69 | 0.92 | | 0.38 |
| Following up the wellbeing of the livestock | 1.02 | 0.90 | | 0.51 | 1.51 | 0.92 | | 0.37 |
| Availing water for the livestock or taking them to water | 0.95 | 0.89 | | 0.53 | 1.42 | 0.90 | | 0.41 |
| Looking after livestock during the day | 0.93 | 0.87 | | 0.41 | 1.48 | 0.92 | | 0.37 |
| Tethering and untethering livestock | 0.92 | 0.89 | | 0.54 | 1.44 | 0.89 | | 0.36 |
| Cutting grass | 1.23 | 0.89 | | 0.48 | 1.65 | 0.91 | | 0.39 |
| Splitting firewood | 1.56 | 0.79 | | 0.41 | 1.93 | 0.81 | | 0.36 |
| Going to market | 1.26 | 0.78 | | 0.36 | 1.68 | 0.76 | | 0.48 |
| Travelling for one hour | 1.03 | 0.71 | | 0.46 | 1.25 | 0.60 | | 0.41 |
| Raising chickens | 0.92 | 0.88 | | 0.62 | 1.22 | 0.89 | | 0.35 |
| Preparing food/ Cooking | 1.05 | 0.88 | | 0.50 | 1.18 | 0.91 | | 0.55 |
| Preparing coffee | 0.87 | 0.81 | | 0.57 | 1.11 | 0.92 | | 0.39 |
| Cleaning house | 0.88 | 0.85 | | 0.62 | 1.18 | 0.91 | | 0.33 |
| Cleaning, cooking and serving utensils | 0.87 | 0.86 | | 0.32 | 1.17 | 0.90 | | 0.37 |
| Able to keep cooking and serving utensils in order | 0.83 | 0.87 | | 0.58 | 1.14 | 0.90 | | 0.02 |
| Fetching water | 0.81 | 0.79 | | 0.54 | 1.08 | 0.88 | | 0.14 |
| Going to mill house to get grain ground | 1.29 | 0.77 | | 0.32 | 1.53 | 0.86 | | 0.64 |
| Washing clothes of the household | 1.21 | 0.88 | | 0.52 | 1.39 | 0.88 | | 0.62 |
| Cleaning the animal area | 1.27 | 0.88 | | 0.60 | 1.29 | 0.91 | | 0.49 |
| Preparing local beverages for the household | 1.29 | 0.81 | | 0.60 | 1.50 | 0.87 | | 0.70 |
| Doing handicraft | 1.23 | 0.85 | | 0.70 | 1.51 | 0.86 | | 0.68 |
| Social Functioning | | | | |  | | | |
| Following up children’s health | 0.88 | 0.81 | 0.47 | | 1.35 | 0.80 | 0.30 | |
| Entertaining or playing with children | 0.80 | 0.81 | 0.49 | | 1.22 | 0.81 | 0.22 | |
| Motivating and encouraging children in their education and other activities | 0.83 | 0.84 | 0.40 | | 1.32 | 0.85 | 0.35 | |
| Communicating well (living in peace and agreement) with family | 0.79 | 0.74 | | 0.41 | 1.44 | 0.65 | 0.55 | |
| Discussing family issues with family members | 0.92 | 0.78 | | 0.40 | 1.58 | 0.78 | 0.42 | |
| Helping parents | 0.99 | 0.82 | | 0.43 | 1.65 | 0.84 | 0.43 | |
| Maintaining social contact with relatives | 0.95 | 0.81 | | 0.50 | 1.39 | 0.80 | 0.46 | |
| Following up children’s hygiene | 0.87 | 0.85 | | 0.33 | 1.47 | 0.86 | 0.42 | |
| Advising and disciplining children | 0.84 | 0.89 | | 0.32 | 1.51 | 0.85 | 0.49 | |
| Feeding children | 0.80 | 0.89 | | 0.48 | 1.21 | 0.87 | 0.53 | |
| Supporting children in wearing clothes | 0.85 | 0.92 | | 0.35 | 1.19 | 0.89 | 0.60 | |
| Changing children’s clothes on time | 0.85 | 0.86 | | 0.69 | 1.22 | 0.89 | 0.51 | |
| Washing children (keeping children’s hygiene) | 0.83 | 0.85 | | 0.64 | 1.22 | 0.89 | 0.56 | |
| Able to keep children from danger | 0.64 | 0.83 | | 0.50 | 1.16 | 0.85 | 0.45 | |
| Communicating well (living in peace and harmony) with neighbors | 0.70 | 0.79 | | 0.53 | 1.08 | 0.72 | 0.53 | |
| Attending coffee ceremonies with neighbors | 0.70 | 0.76 | | 0.50 | 1.11 | 0.74 | 0.51 | |
| Doing different tasks in cooperation with neighbors | 0.99 | 0.84 | | 0.41 | 1.53 | 0.88 | 0.23 | |
| Giving practical support to neighbors when there is mourning or any  other ceremony | 1.12 | 0.80 | | 0.35 | 1.60 | 0.88 | 0.23 | |

| Item | Service users (N=200) | | | Caregivers (N=200) | | |
| --- | --- | --- | --- | --- | --- | --- |
|  | **Mean** | **Item-scale correlation** | **ICC** | **Mean** | **Item-scale correlation** | **ICC** |
| Going and attending when there is mourning in the neighborhood | 1.02 | 0.85 | 0.41 | 1.52 | 0.81 | 0.29 |
| Contributing to Keep security/peace in the neighborhood | 1.01 | 0.89 | 0.42 | 1.56 | 0.90 | 0.37 |
| Communicating well (living in peace and harmony) with the community | 0.77 | 0.81 | 0.49 | 1.18 | 0.77 | 0.18 |
| Talking (enjoying) with friends | 0.72 | 0.80 | 0.66 | 1.20 | 0.79 | 0.33 |
| Participating in “Idir” | 1.06 | 0.88 | 0.54 | 1.62 | 0.87 | 0.34 |
| Going to weddings, funerals, baptism, and other ceremonies | 1.09 | 0.86 | 0.55 | 1.68 | 0.86 | 0.28 |
| Visiting postnatal women, people who are sick, prisoners and elderly | 1.02 | 0.87 | 0.41 | 1.66 | 0.90 | 0.42 |
| Participating in and preparing Mahiber/Senbete/Lika/Dado | 1.13 | 0.83 | 0.44 | 1.75 | 0.83 | 0.46 |
| Attending Kebele and village meetings | 1.21 | 0.84 | 0.57 | 1.80 | 0.87 | 0.44 |
| Going to church/mosque | 1.03 | 0.74 | 0.26 | 1.54 | 0.75 | 0.36 |
| Praying (doing “selat”) | 1.12 | 0.69 | 0.22 | 1.51 | 0.75 | 0.40 |
| Giving food or money for those who are in need | 0.79 | 0.79 | 0.42 | 1.49 | 0.84 | 0.33 |
| Participating in, supporting and coordinating religious celebrations | 0.95 | 0.88 | 0.37 | 1.61 | 0.83 | 0.46 |

*Intra-class correlation coefficient

**Additional file 3. Mean value, item –total correlation and alpha value of items in the validation study.**

| Item | Service users (N=150) | | | Caregivers (N=150) | | |
| --- | --- | --- | --- | --- | --- | --- |
|  | **Mean** | **Item-scale correlation** | **Alpha** | **Mean** | **Item-scale correlation** | **Alpha** |
| Self-care | | | |  | | |
| Able to ask for or prepare and eat food when needed | 1.08 | 0.53 | 0.95 | 1.49 | 0.67 | 0.95 |
| Washing own body | 0.92 | 0.81 | 0.94 | 1.54 | 0.88 | 0.94 |
| Washing hands before and after eating | 0.70 | 0.84 | 0.93 | 1.25 | 0.82 | 0.94 |
| Washing own clothes | 1.10 | 0.83 | 0.93 | 1.80 | 0.81 | 0.94 |
| Cutting nails | 0.81 | 0.83 | 0.93 | 1.57 | 0.85 | 0.94 |
| Able to change clothes when it gets dirty | 0.97 | 0.85 | 0.93 | 1.61 | 0.89 | 0.94 |
| Able to keep oneself from danger | 0.89 | 0.71 | 0.94 | 1.27 | 0.72 | 0.95 |
| Using the toilet properly | 0.64 | 0.77 | 0.94 | 0.83 | 0.68 | 0.95 |
| Washing hair | 0.86 | 0.87 | 0.93 | 1.54 | 0.91 | 0.94 |
| Work | | | |  | | |
| Working in the field | 1.94 | 0.87 | 0.95 | 2.57 | 0.91 | 0.95 |
| Kitchen gardening | 1.88 | 0.90 | 0.95 | 2.59 | 0.92 | 0.94 |
| Looking after and attending livestock during the day | 1.78 | 0.89 | 0.95 | 2.39 | 0.92 | 0.94 |
| Cutting grass | 1.87 | 0.91 | 0.95 | 2.50 | 0.90 | 0.95 |
| Splitting firewood | 1.92 | 0.88 | 0.95 | 2.51 | 0.91 | 0.94 |
| Going to market | 1.73 | 0.81 | 0.96 | 2.34 | 0.85 | 0.95 |
| Travelling for one hour | 1.55 | 0.75 | 0.96 | 1.86 | 0.57 | 0.97 |
| Raising chickens | 1.46 | 0.80 | 0.96 | 2.01 | 0.86 | 0.99 |
| Preparing food/ Cooking | 1.51 | 0.89 | 0.95 | 2.22 | 0.95 | 0.98 |
| Cleaning house | 1.38 | 0.91 | 0.95 | 2.12 | 0.94 | 0.98 |
| Going to mill house to get grain ground | 1.80 | 0.84 | 0.73 | 2.29 | 0.97 | 0.98 |
| Washing clothes of the household | 1.52 | 0.89 | 0.89 | 2.25 | 0.94 | 0.98 |
| Cleaning the animal area | 1.57 | 0.90 | 0.90 | 2.26 | 0.93 | 0.98 |
| Preparing local beverages for the household | 1.48 | 0.82 | 0.82 | 2.28 | 0.94 | 0.98 |
| Doing handicraft | 1.81 | 0.74 | 0.74 | 2.35 | 0.93 | 0.98 |
| Social functioning | | | |  | | |
| Following up children’s health | 1.42 | 0.80 | 0.98 | 2.08 | 0.86 | 0.98 |
| Motivating and encouraging children in their education and other activities | 1.41 | 0.84 | 0.98 | 2.07 | 0.87 | 0.98 |
| Communicating well (living in peace and agreement) with family | 1.49 | 0.74 | 0.98 | 2.23 | 0.72 | 0.98 |
| Discussing family issues with family members | 1.60 | 0.85 | 0.98 | 2.45 | 0.89 | 0.98 |
| Helping parents or close elderly relatives | 1.56 | 0.88 | 0.98 | 2.28 | 0.90 | 0.98 |
| Maintaining social contact with relatives | 1.51 | 0.84 | 0.98 | 2.17 | 0.87 | 0.98 |
| Following up children’s hygiene | 1.44 | 0.91 | 0.97 | 2.21 | 0.91 | 0.98 |
| Advising and disciplining children | 1.47 | 0.91 | 0.97 | 2.28 | 0.92 | 0.98 |
| Communicating well (living in peace and harmony) with neighbors | 1.23 | 0.79 | 0.98 | 2.06 | 0.80 | 0.98 |
| Doing different tasks in cooperation with neighbors | 1.49 | 0.88 | 0.97 | 2.33 | 0.89 | 0.98 |
| Going and attending when there is mourning in the neighborhood | 1.54 | 0.87 | 0.97 | 2.32 | 0.89 | 0.98 |
| Participating in “Idir” | 1.61 | 0.79 | 0.98 | 2.18 | 0.86 | 0.98 |
| Visiting postnatal women, people who are sick, prisoners and elderly | 1.51 | 0.88 | 0.97 | 2.16 | 0.87 | 0.98 |
| Participating in and preparing Mahiber/Senbete/Lika/Dado | 1.65 | 0.78 | 0.98 | 2.20 | 0.85 | 0.98 |
| Attending Kebele and village meetings | 1.65 | 0.85 | 0.98 | 2.33 | 0.85 | 0.98 |
| Going to church/mosque | 1.50 | 0.73 | 0.98 | 2.13 | 0.83 | 0.98 |
| Giving food or money for those who are in need | 1.33 | 0.79 | 0.98 | 2.14 | 0.81 | 0.98 |

**Additional file 4. Pearson’s correlation between WHODAS and the BFS (n=150): service users (caregivers).**

|  | WHODAS** | | | | | | | |
| --- | --- | --- | --- | --- | --- | --- | --- | --- |
| BFS* | Cognition | Mobility | Self-care | Getting along | HH*** activities | Work | Participation | WHODAS |
| Self-care | 0.64(0.69) | 0.55(0.36) | 0.77(0.81) | 0.64(0.63) | 0.59(0.63) | 0.57(0.64) | 0.59(0.63) | 0.70(0.73) |
| Work (men and women) | 0.76(0.80) | 0.65(0.56) | 0.67(0.60) | 0.66(0.65) | 0.76(0.84) | 0.74(0.85) | 0.72(0.73) | 0.80(0.84) |
| Work (women only) | 0.72(0.63) | 0.67(0.50) | 0.68(0.51) | 0.63(0.54) | 0.70(0.69) | 0.68(0.68) | 0.68(0.56) | 0.77(0.68) |
| Social functioning | 0.81(0.88) | 0.64(0.45) | 0.77(0.72) | 0.76(0.81) | 0.78(0.81) | 0.75(0.82) | 0.79(0.79) | 0.86(0.89) |
| Overall scale | 0.82(0.86) | 0.70(0.51) | 0.81(0.76) | 0.76(0.77) | 0.79(0.83) | 0.77(0.83) | 0.79(0.77) | 0.88(0.89) |

*Butajira Functioning Scale; **WHO Disability Assessment Schedule; ***House hold

### Additional file 5. Exploratory factor analysis of the piloting data.

*Factor loadings of the self-care items*

| Item | Service users | | Caregivers | |
| --- | --- | --- | --- | --- |
|  | **Factor 1** | **Factor 2** | **Factor 1** | **Factor 2** |
| Able to eat food in a proper manner | 0.25 | 0.93 | 0.21 | 0.93 |
| Able to eat food on time | 0.33 | 0.84 | 0.38 | 0.81 |
| Washing own body | 0.68 | 0.35 | 0.78 | 0.33 |
| Washing hands before and after eating | 0.73 | 0.38 | 0.73 | 0.41 |
| Washing own clothes | 0.67 | 0.39 | 0.84 | 0.24 |
| Cutting nails | 0.75 | 0.35 | 0.91 | 0.24 |
| Washing hair | 0.88 | 0.29 | 0.94 | 0.24 |
| Getting haircut (Getting hair dressed) | 0.83 | 0.33 | 0.92 | 0.28 |
| Brushing teeth | 0.85 | 0.32 | 0.89 | 0.30 |
| Able to change clothes when it gets dirty | 0.71 | 0.24 | 0.81 | 0.25 |
| Able to keep oneself from danger | 0.57 | 0.32 | 0.62 | 0.41 |
| Using the toilet properly | 0.58 | 0.34 | 0.49 | 0.31 |

*Factor loadings of men and women shared work items*

| Item | Service users | Caregivers |
| --- | --- | --- |
|  | **Factor 1** | **Factor 1** |
| Working in the field (ploughing, reaping) | 0.77 | 0.75 |
| Working in the field (weeding, digging, threshing, cleaning land for threshing) | 0.79 | 0.76 |
| Kitchen gardening | 0.86 | 0.88 |
| Collecting grass and straw for livestock food | 0.94 | 0.93 |
| Following up the wellbeing of the livestock | 0.95 | 0.96 |
| Availing water for the livestock or taking them to water | 0.94 | 0.95 |
| Looking after livestock during the day | 0.92 | 0.97 |
| Tethering and untethering livestock | 0.93 | 0.94 |
| Cutting grass | 0.87 | 0.93 |
| Splitting firewood | 0.75 | 0.80 |
| Going to market | 0.76 | 0.75 |
| Travelling for one hour | 0.73 | 0.61 |

*Factor loadings of women only work items*

| Item | Service users | | Caregivers | |
| --- | --- | --- | --- | --- |
|  | **Factor 1** | **Factor 2** | **Factor 1** | **Factor 2** |
| Working in the field (ploughing, reaping) | 0.29 | 0.89 | 0.35 | 0.73 |
| Working in the field (weeding, digging, threshing, cleaning land for threshing) | 0.27 | 0.90 | 0.38 | 0.72 |
| Kitchen gardening | 0.40 | 0.86 | 0.49 | 0.73 |
| Collecting grass and straw for livestock food | 0.51 | 0.80 | 0.37 | 0.86 |
| Following up the wellbeing of the livestock | 0.63 | 0.67 | 0.53 | 0.74 |
| Availing water for the livestock or taking them to water | 0.63 | 0.68 | 0.61 | 0.66 |
| Looking after livestock during the day | 0.63 | 0.63 | 0.55 | 0.76 |
| Tethering and untethering livestock | 0.66 | 0.65 | 0.67 | 0.62 |
| Cutting grass | 0.44 | 0.77 | 0.51 | 0.77 |
| Splitting firewood | 0.42 | 0.73 | 0.47 | 0.64 |
| Going to market | 0.55 | 0.59 | 0.50 | 0.64 |
| Travelling for one hour | 0.56 | 0.42 | 0.48 | 0.44 |
| Raising chickens | 0.76 | 0.51 | 0.64 | 0.64 |
| Preparing food/ Cooking | 0.71 | 0.53 | 0.82 | 0.44 |
| Preparing coffee | 0.84 | 0.30 | 0.88 | 0.40 |
| Cleaning house | 0.86 | 0.36 | 0.86 | 0.41 |
| Cleaning cooking and serving utensils | 0.82 | 0.44 | 0.92 | 0.33 |
| Able to keep cooking and serving utensils in order | 0.85 | 0.42 | 0.91 | 0.34 |
| Fetching water | 0.82 | 0.29 | 0.83 | 0.39 |
| Going to mill house to get grain ground | 0.55 | 0.49 | 0.70 | 0.50 |
| Washing clothes of the household | 0.64 | 0.59 | 0.80 | 0.42 |
| Cleaning the animal area | 0.62 | 0.63 | 0.75 | 0.53 |
| Preparing local beverages for the household (Tela/Keribu/Kinato) | 0.59 | 0.51 | 0.67 | 0.53 |
| Doing handicraft (such as “kasha”, “mosob”, and “dantel”) | 0.63 | 0.56 | 0.63 | 0.56 |

*Factor loadings of men and women shared social functioning items*

| Item | Service users | | Caregivers | |
| --- | --- | --- | --- | --- |
|  | **Factor 1** | **Factor 2** | **Factor 1** | **Factor 2** |
| Following up children’s health | 0.35 | 0.84 | 0.59 | 0.56 |
| Entertaining or playing with children | 0.31 | 0.89 | 0.50 | 0.66 |
| Motivating and encouraging children in their education and other activities | 0.39 | 0.84 | 0.53 | 0.68 |
| Communicating well (living in peace and agreement) with family | 0.41 | 0.64 | 0.17 | 0.81 |
| Discussing family issues with family members | 0.44 | 0.67 | 0.31 | 0.86 |
| Helping parents | 0.45 | 0.74 | 0.44 | 0.80 |
| Maintaining social contact with relatives | 0.51 | 0.63 | 0.43 | 0.73 |
| Following up children’s hygiene | 0.42 | 0.83 | 0.49 | 0.77 |
| Advising and disciplining children | 0.49 | 0.80 | 0.49 | 0.76 |
| Communicating well (living in peace and harmony) with neighbors | 0.70 | 0.41 | 0.47 | 0.54 |
| Attending coffee ceremonies with neighbors | 0.68 | 0.40 | 0.52 | 0.51 |
| Doing different tasks in cooperation with neighbors | 0.71 | 0.48 | 0.66 | 0.59 |
| Giving practical support to neighbors when there is mourning or any other ceremony | 0.75 | 0.38 | 0.69 | 0.57 |
| Going and attending when there is mourning in the neighborhood | 0.76 | 0.44 | 0.68 | 0.47 |
| Contributing to Keep security/peace in the neighborhood | 0.74 | 0.52 | 0.66 | 0.63 |
| Communicating well (living in peace and harmony) with the community | 0.72 | 0.41 | 0.59 | 0.47 |
| Talking (enjoying) with friends | 0.74 | 0.38 | 0.65 | 0.45 |
| Participating in “Idir” | 0.75 | 0.51 | 0.73 | 0.50 |
| Going to weddings, funerals, baptism, and other ceremonies | 0.77 | 0.46 | 0.78 | 0.44 |
| Visiting postnatal women, people who are sick, prisoners and elderly | 0.77 | 0.45 | 0.83 | 0.44 |
| Participating in and preparing Mahiber/Senbete/Lika/Dado | 0.73 | 0.44 | 0.82 | 0.36 |
| Attending Kebele and village meetings | 0.72 | 0.47 | 0.84 | 0.40 |
| Going to church/mosque | 0.61 | 0.42 | 0.80 | 0.23 |
| Praying (doing “selat”) | 0.59 | 0.38 | 0.77 | 0.26 |
| Giving food or money for those who are in need | 0.62 | 0.51 | 0.74 | 0.43 |
| Participating in, supporting and coordinating religious celebrations | 0.69 | 0.55 | 0.79 | 0.36 |

*Factor loadings of women only social functioning items*

| Item | Service users | | Caregivers | |
| --- | --- | --- | --- | --- |
|  | **Factor 1** | **Factor 2** | **Factor 1** | **Factor 2** |
| Following up children’s health | 0.87 | 0.40 | 0.69 | 0.49 |
| Entertaining or playing with children | 0.84 | 0.37 | 0.71 | 0.48 |
| Motivating and encouraging children in their education and other activities | 0.83 | 0.43 | 0.62 | 0.56 |
| Communicating well (living in peace and agreement) with family | 0.84 | 0.37 | 0.61 | 0.20 |
| Discussing family issues with family members | 0.87 | 0.40 | 0.68 | 0.36 |
| Helping parents | 0.80 | 0.41 | 0.71 | 0.44 |
| Maintaining social contact with relatives | 0.84 | 0.40 | 0.51 | 0.57 |
| Following up children’s hygiene | 0.84 | 0.40 | 0.79 | 0.44 |
| Advising and disciplining children | 0.86 | 0.45 | 0.75 | 0.47 |
| Feeding children | 0.67 | 0.60 | 0.87 | 0.36 |
| Supporting children in wearing clothes | 0.65 | 0.67 | 0.93 | 0.34 |
| Changing children’s clothes on time | 0.65 | 0.58 | 0.92 | 0.35 |
| Washing children (keeping children’s hygiene) | 0.63 | 0.59 | 0.88 | 0.38 |
| Able to keep children from danger | 0.57 | 0.61 | 0.74 | 0.46 |
| Communicating well (living in peace and harmony) with neighbors | 0.49 | 0.62 | 0.56 | 0.51 |
| Attending coffee ceremonies with neighbors | 0.46 | 0.57 | 0.54 | 0.51 |
| Doing different tasks in cooperation with neighbors | 0.57 | 0.70 | 0.56 | 0.67 |
| Giving practical support to neighbors when there is mourning or any other ceremony | 0.44 | 0.78 | 0.46 | 0.73 |
| Going and attending when there is mourning in the neighborhood | 0.45 | 0.78 | 0.40 | 0.70 |
| Contributing to Keep security/peace in the neighborhood | 0.60 | 0.70 | 0.58 | 0.72 |
| Communicating well (living in peace and harmony) with the community | 0.62 | 0.60 | 0.49 | 0.60 |
| Talking (enjoying) with friends | 0.49 | 0.75 | 0.46 | 0.70 |
| Participating in “Idir” | 0.47 | 0.78 | 0.48 | 0.70 |
| Going to weddings, funerals, baptism, and other ceremonies | 0.37 | 0.83 | 0.37 | 0.79 |
| Visiting postnatal women, people who are sick, prisoners and elderly | 0.51 | 0.76 | 0.41 | 0.83 |
| Participating in and preparing Mahiber/Senbete/Lika/Dado | 0.53 | 0.65 | 0.37 | 0.79 |
| Attending Kebele and village meetings | 0.48 | 0.72 | 0.43 | 0.83 |
| Going to church/mosque | 0.45 | 0.56 | 0.25 | 0.84 |
| Praying (doing “selat”) | 0.39 | 0.62 | 0.38 | 0.77 |
| Giving food or money for those who are in need | 0.50 | 0.63 | 0.49 | 0.67 |
| Participating in, supporting and coordinating religious celebrations | 0.56 | 0.64 | 0.46 | 0.75 |

**Additional file 6. English version of the Butajira Functioning Scale (BFS)**

| **In the past one month, how much difficulty did you have in accomplishing the following tasks/activities compared with most other people of your age and sex?** |
| --- |

| **S.N** | **Activity/Task** | | **Degree of difficulty accomplishing the task/activity** | | | | | |
| --- | --- | --- | --- | --- | --- | --- | --- | --- |
|  |  | | None | Little | Moderate | A lot | Can’t do task | Not applicable |
| 1. **Self-care** | | | | | | | | |
| 1 | Able to ask for or prepare and eat food when needed | |  |  |  |  |  |  |
| 2 | Washing own body | |  |  |  |  |  |  |
| 3 | Washing hands before and after eating | |  |  |  |  |  |  |
| 4 | Washing own clothes | |  |  |  |  |  |  |
| 5 | Cutting nails | |  |  |  |  |  |  |
| 6 | Able to change clothes when it gets dirty | |  |  |  |  |  |  |
| 7 | Able to keep oneself from danger | |  |  |  |  |  |  |
| 8 | Using the toilet properly [USETOILT] | |  |  |  |  |  |  |
| 9 | Washing hair | |  |  |  |  |  |  |
| 1. **Work** | | | | | | | | |
| 10 | Working in the field | |  |  |  |  |  |  |
| 11 | Kitchen gardening | |  |  |  |  |  |  |
| 12 | Looking after and attending livestock during the day | |  |  |  |  |  |  |
| 13 | Cutting grass | |  |  |  |  |  |  |
| 14 | Splitting firewood | |  |  |  |  |  |  |
| 15 | Going to market | |  |  |  |  |  |  |
| 16 | Travelling for one hour | |  |  |  |  |  |  |
| 17 | Raising chickens | |  |  |  |  |  |  |
| 18 | Preparing food/ Cooking | |  |  |  |  |  |  |
| 19 | Cleaning house | |  |  |  |  |  |  |
| 20 | Going to mill house to get grain ground | |  |  |  |  |  |  |
| 21 | Washing clothes of the household | |  |  |  |  |  |  |
| 22 | Cleaning the animal area | |  |  |  |  |  |  |
| 23 | Preparing local beverages for the household (Tela/Keribu/Kinato) | |  |  |  |  |  |  |
| 24 | Doing handicraft (such as “kasha”, “mosob”, and “dantel”) | |  |  |  |  |  |  |
| 1. **Social Functioning** | | | | | | | | |
| 25 | Following up children’s health | |  |  |  |  |  |  |
| 26 | Motivating and encouraging children in their education and other activities | |  |  |  |  |  |  |
| 27 | Communicating well (living in peace and agreement) with family | |  |  |  |  |  |  |
| 28 | Discussing family issues with family members | |  |  |  |  |  |  |
| 29 | Helping parents or close elderly relatives | |  |  |  |  |  |  |
| 30 | Maintaining social contact with relatives | |  |  |  |  |  |  |
| 31 | Following up children’s hygiene | |  |  |  |  |  |  |
| 32 | Advising and disciplining children | |  |  |  |  |  |  |
| 33 | Communicating well (living in peace and harmony) with neighbors | |  |  |  |  |  |  |
| 34 | Doing different tasks in cooperation with neighbors | |  |  |  |  |  |  |
| 35 | Going and attending when there is mourning in the neighborhood | |  |  |  |  |  |  |
| 36 | Participating in “Idir” | |  |  |  |  |  |  |
| 37 | Visiting postnatal women, people who are sick, prisoners and elderly | |  |  |  |  |  |  |
| 38 | Participating in and preparing Mahiber/Senbete/Lika/Dado | |  |  |  |  |  |  |
| 39 | Attending Kebele and village meetings | |  |  |  |  |  |  |
| 40 | | Going to church/mosque |  |  |  |  |  |  |
| 41 | | Giving food or money for those who are in need |  |  |  |  |  |  |
